# Chicago Pathological Society

**Published:** 1888-07

**Authors:** 


### SOCIETY REPORTS.

### CHICAGO PATHOLOGICAL SOCIETY.

### JUNE MEETING.

President I. N. Danforth, in the chair.

Dr. Albert B. Strong read a paper on "CALCULI IN THE BLADDER OR URETHRA OF CHILDREN."

It is said that in those regions of Germany where sour wine is freely consumed the drinkers mix the beverage with an alkali to guard against kidney colic. Dr. E. Powell reports the case of a physician who invariably suffers from renal colic within the next twenty-four hours after drinking a glass of sour wine. One or two days afterward he always passes from the urethra a uric acid calculus as long as a grain of wheat.

The author has during the past fifteen years seen seventeen cases of calculi in children varying in age from one to eight years. Of these cases three were operated upon by the left lateral perineal section and the stone removed. In ten cases the stone was passed spontaneously, or with assistance, through the urethra. In four cases an operation was refused, and the patients died from uræmia.

Of these cases two were children of German and fifteen of Polish parents. This large proportion of stone cases occurring among children of one nationality excited very great interest, and the reason was accordingly eagerly sought for. The large Polish population of the northwestern portion of this city furnished the cases. In the course of the investigations many other cases of calculi in the bladder or urethra were heard of, but not personally seen. These people are prolific, and it is not at all uncommon to see a family of eight or ten children. As a rule the patients are very poor and their sanitary surroundings most unfavorable.

Among these people, as a rule, the mother nurses the baby until it is ten or twelve months of age, when pregnancy interrupts lactation, and the child subsequently subsists largely upon sausage, put up in small gut-cases. This article is cheap, easily prepared, and very nutritious, and the little one sucks and munches his food several times a day. Carpenter says: "On a full meat diet the normal daily excretion of ten grains of uric acid is increased to 32.5 grains." Uric acid forms the nucleus of fully 95 per centum of all urinary calculi. Therefore in these cases reported it seems reasonable to infer that meat diet is the most potent etiological factor. In these cases no examination was made for the nucleus of the stones which were exhibited. The smallest weighed two grains, and was from a child twenty-two months of age. The next in size weighed eight grains, and was from a child twenty-seven months old. The largest weighed three and one half drachms, and was removed from a boy eight years of age.

Two points in the history of the latter case are interesting:

(1) The renal origin of the stone, and

(2) The years of dribbling of urine which followed the operation.

The mother gives the following history: "The child was perfectly well up to three years of age, when he had a mild attack of measles. On the third day of the eruption the boy suddenly sprang out of bed, complaining of great pain in the lower part of the abdomen. He screamed and ran about the room, and continually crying out, 'Oh, my belly!' This severe attack lasted an hour, and milder attacks were of frequent occurrence for the next four days, during which time he frequently pulled at his penis. His urine came in drops, and he appeared to suffer much pain in voiding it. On the fourth day the pain and habit of pulling at the penis suddenly disappeared, and he remained in perfect health for the subsequent six months, when he was seized with a second attack, similar to the first, which also lasted three or four days. Again the trouble disappeared as suddenly as it had appeared. He was then perfectly well for four months, when a third attack seized him. During the last three years these attacks have been of frequent occurrence, while the habit of pulling at the penis continued more or less constantly; for half an hour after urinating the child would scream with pain. Ten days before the operation here reported he was seized with excruciating pain which has been paroxysmal ever since. During the paroxysm the child was perfectly uncontrollable, throwing his body about in violent contortions. This condition terminated in a spasm on the tenth day. During the ten days the patient complained of pain shooting from the right lumbar region downward toward the bladder. He also suffered great pain at the head of the penis."

There are a few points which admit of special mention in this case. The repeated attacks of renal colic demonstrate the origin of the stone. There was no history of excessive meat diet. Again, the patient had been seen within the last day or two prior to this report, and the author learned that for the first five years succeeding the operation there was constant dribbling of urine during the daytime. Yet during the next three years the condition was present only when the boy caught cold. Since then he has been perfectly well in this respect. He is now a large, well-developed boy, following the laborious work of a molder.

This dribbling of urine after perineal lithotomy is not alone confined to this case. Other operators say that it is not at all uncommon. The text-books consulted are, however, silent on this point. In giving a prognosis in these cases, this possible condition should not be forgotten.

Concerning perineal section in children, there are a few peculiarities dependent upon the different anatomical relations and nature of the infantile bladder. Such a bladder, aside from its small size, is differently situated in the pelvis. It is much higher, so that the distance from the skin to the base of the organ is relatively greater than in the adult. As a result of this anatomical peculiarity, in opening the bladder the handle of the knife must be depressed more than in the adult, otherwise there is danger of cutting into the space between this viscus and the rectum.

Again, the bulb of the urethra and the prostate gland are so rudimentary that neither practically exists, hence there is little danger of wounding the bulb or its artery. Again, the recto-vesicle fold of peritoneum is much higher than in the adult, and consequently there is but little danger of opening the peritoneal cavity should the incision be extended back unduly far.

The advice given in the recent edition of the Encyclopedia of Surgery, to cut freely through the prostate and base of the bladder should be condemned.

Dr. Venn extracts urethral calculi when they are lodged in the canal anterior to the perineum by an ingenious method. He distends the canal in front of the obstruction with oil, and then the stone is easily manipulated forward, and, if necessary, removed with the forceps.

Dr. Strong had recently removed a stone from a boy seventeen years of age, which weighed one ounce and a half.

Dr. Skeer reported a case of stone which had lodged in the prostate gland. It gave him a great deal of trouble, and was three-quarters of an inch long and half an inch in diameter. He also mentioned the case of a lady who passed a stone from the urethra as large as the seed of a prune.

Professor E. Root asked whether the patients were troubled with nocturnal incontinence, to which Dr. Strong replied that all of them were. Incontinence was an early symptom in most of the cases, and the next most prominent one was pulling at the penis.

Dr. Strong had never met with calculi in girls, but had read of such cases..

Professor Silva knew of a lady, married, 30 to 35 years of age, who had spontaneously passed a calculus, after intense suffering, as large as the largest exhibited by Dr. Strong. It was pear-shaped. A peculiarity in connection with the case was that at the time she was considered to be in labor.

